# First mitogenome of *Anthomyia illocata* (Diptera, Anthomyiidae) yielded by next-generation sequencing

**DOI:** 10.1080/23802359.2022.2076626

**Published:** 2022-05-26

**Authors:** Henan Li, Liping Yan, Wenya Pei, Yang Hu, Aidong Wang, Zhiyuan Wang, Dong Zhang

**Affiliations:** a School of Ecology and Nature Conservation, Beijing Forestry University, Beijing, China; b Forest Protection Station of Tongzhou District, Beijing, China

**Keywords:** Anthomyiidae, Calyptratae, mitochondrial genome, phylogeny

## Abstract

The mitochondrial genome of *Anthomyia illocata* Walker, 1857 belonging to the Anthomyiidae, was obtained using a next-generation sequencing approach. This 16,236 bp complete mitogenome consists of 13 protein-coding, two ribosomal RNA, and 22 transfer RNA genes, as well as a non-coding control region. The Anthomyiidae are reconstructed as a paraphyletic group, with the genera *Pegomya* recovered as a sister group of the Scathophagidae.

The family Anthomyiidae belongs to Calyptratae (Diptera: Schizophora), with approximately 2000 described species from 40 genera all over the world (Grisales et al. 2016). Many anthomyiids are active pollinators and usually inhabit moist, cold forests, while others feed on fermented vegetation or animal urine and feces. *Anthomyia illocata* larvae are coprophagous and adults live in different types of dung and carrion, may act as potential vectors for the spread of many diseases to different environments (Michelsen 2010). Mitogenomic data have been widely used in studies of phylogeny and evolution (Zhang et al. 2016; Yan et al. 2017, 2019), as well as population genetics, even in the era of phylogenomics (Kutty et al. 2019; Yan et al. 2021). There are currently mitogenomes of seven Anthomyiidae species available in GenBank. Here, we sequenced mitochondrial genome of *Anthomyia illocata* Walker, 1857, representing the first mitochondrial genome of the *Anthomyia*, to investigate the genera-level relationship of Anthomyiidae, which will help us to understand the evolutionary history and contribute to future genetic research of this family.

The adult specimen broad, dark, postsutural transverse band on thorax, scutellum anteriorly black and posteriorly gray of *A*. *illocata* used for DNA extraction was collected on 13 July 2015 from Panzhihua of Sichuan, China (26°36′46"N, 101°35′21"E), and deposited at the Museum of Beijing Forestry University, Beijing, China (http://bjfc.bjfu.edu.cn, contact person: Henan Li, email: 1377903058@qq.com) under the voucher number BFU RNA-113. The DNA sample was deposited at the Laboratory of Animal Noninvasive Studies on School of Ecology and Nature Conservation, Beijing Forestry University, Beijing, China. The genomic DNA was extracted with the muscle tissues of the thorax of an adult using the DNeasy Blood and Tissue kit (QIAGEN Sciences, Valencia, CA). The genomic DNA was pooled with other insect species and sequenced using the Illumina NovaSeq 6000 (PE150, Illumina, San Diego, CA) platform. A total of 4 Gb raw data were generated and trimmed using Trimmomatic (Bolger et al. 2014) before assembling with idba_ud implemented with IDBA-1.1.1 (Peng et al. 2012). The similarity of alignment for assembling was set to be 0.98. Mitogenome was then pulled out using a Blast search (Altschul et al. 1990) with *COI* as bait sequence (Crampton-Platt et al. 2015). Protein-coding genes (PCGs) and ribosomal RNA genes were annotated by aligning with the homologous genes reported in other calyptrate flies (Zhang et al. 2016). Transfer RNA genes were identified using MITOS webserver with invertebrate genetic code (Bernt et al. 2013).

The complete mitochondrial genome of *A. illocata* is 16,236 bp in length and contains 13 PCGs, two ribosomal RNA genes, 22 transfer RNA genes, and a non-coding control region. The genome sequence data that support the findings of this study are openly available in GenBank of NCBI at https://www.ncbi.nlm.nih.gov/nuccore/MW296030 under the accession no. MW296030. The associated BioProject, SRA, and Bio-Sample numbers are PRJNA794964, SRS12029158, and SAMN24665691, respectively. The overall nucleotide composition is estimated to be 39.94% of A, 38.84% of T, 12.30% of C, and 8.92% of G, with a slightly higher A + T content (78.7%) than other calyptratae (Agudelo et al. 2019; Tang et al. 2019). Most of the 13 PCGs used ATN as the start codon (ATG for *COII*, *ATP6*, *COIII*, *ND4*, *ND4L*, and *CYTB*; ATT for *ND2*, *ND5*, and *ND6*; ATA for *ND3* and *ND1*; ATC for *ATP8*), except that *COI* begins with codon TCG. The stop codon TAA is assigned to most of the PCGs (*ND2*, *ATP8*, *ATP6*, *COI*, *COIII*, *ND3*, *ND4L*, *ND6*, and *ND1*), but an incomplete stop codon T is used by three PCGs (*COII*, *ND5*, and *ND4*), and *CYTB* terminates with the codon TAG. In total, there were 43 overlaps between neighboring genes at 13 locations, ranging in size from 1 to 8 bp. Excluding the control region, there were 159 intergenic spacers at 15 locations, in stretches ranging from 1 to 62 bp.

Complete mitochondrial genomes of other calyptrate families were harvested from GenBank for phylogenetic analysis, with *Drosophila melanogaster* (Drosophilidae) used to root the tree. Bayesian inference (BI) reconstruction was performed using MrBayes (Ronquist et al. 2012) with dataset containing nucleotide sequences of 13 PCGs aligned with MAFFT (Katoh and Standley 2013). The evolutionary model for each partition was assigned by PartitionFinder 2 (Lanfear et al. 2017). Bayesian inference analysis was performed by running 10 million generations with sampling every 1000 generations. The muscoids were supported as paraphyletic group, with (Anthomyiidae + Scathophagidae) recovered as the sister group to the clade Oestroidea ((Calliphoridae + Sarcophagidae)+Tachinidae) (posterior probabilities = 0.98) (Figure 1), which is consistent with earlier studies (Kutty et al. 2010, 2019; Yan et al. 2021). The Anthomyiidae was inferred as paraphyletic, within which Scathophagidae was nested, and the species *Pegoplata infirma* was recovered as a basal branch of the clade (Anthomyiidae + Scathophagidae). At the genus level, the clade (*Botanophila*+*Fucellia*) formed a sister group of the monotypic genus *Delia* with strong support (posterior probabilities = 0.99). The *Anthomyia*, with mitogenome documented by this study, made sister group to the genera *Hylemya* is represented by *Hylemya vagans* (posterior probabilities = 1).

**Figure 1.**
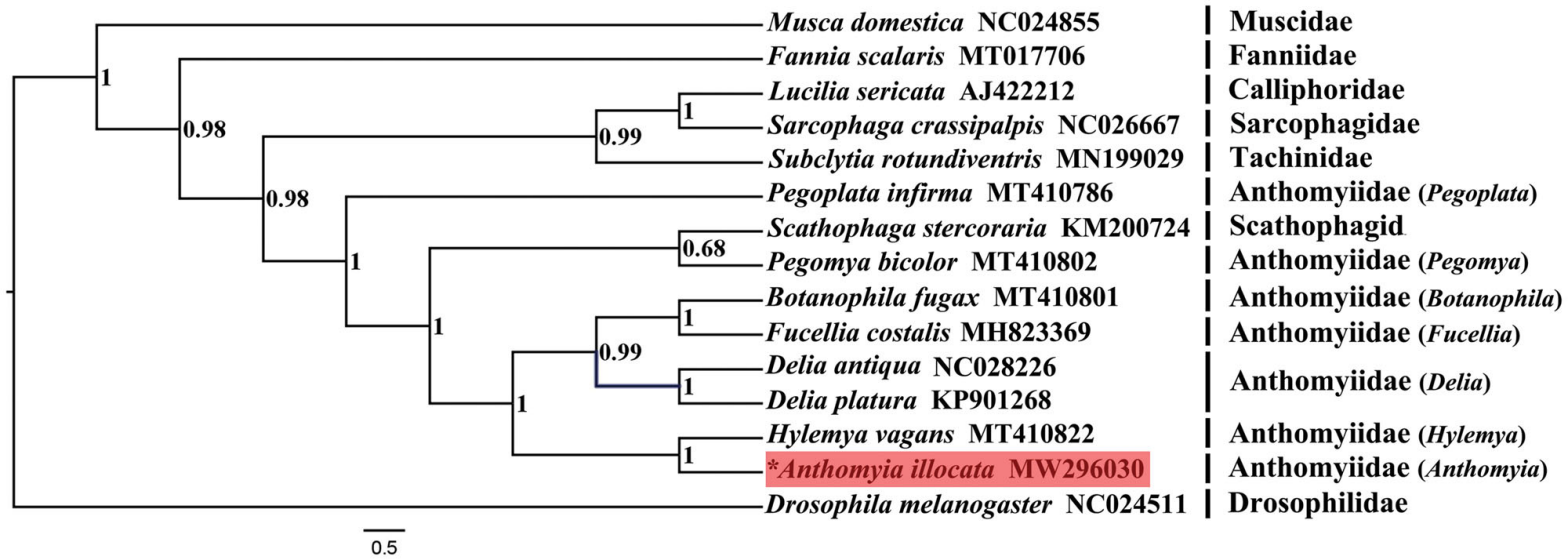
BI phylogenetic tree of 15 species which consists of eight Anthomyiidae species and seven outgroups. *Species documented in this study.

## Data Availability

The genome sequence data that support the findings of this study are openly available in GenBank of NCBI at https://www.ncbi.nlm.nih.gov/nuccore/MW296030 under the accession no. MW296030. The associated BioProject, SRA, and Bio-Sample numbers are PRJNA794964, SRS12029158, and SAMN24665691, respectively.
